# Optical Control of Adenosine-Mediated Pain Modulation

**DOI:** 10.1021/acs.bioconjchem.1c00387

**Published:** 2021-08-27

**Authors:** Katharina Hüll, Víctor Fernández-Dueñas, Matthias Schönberger, Marc López-Cano, Dirk Trauner, Francisco Ciruela

**Affiliations:** † Department of Chemistry, New York University, 100 Washington Square East, New York City, New York 10003, United States; ‡ Department of Chemistry and Center for Integrated Protein Munich, Ludwig-Maximilians-Universität Menchen, Butenandtstrasse 5−13, 81377 Munich, Germany; § Pharmacology Unit, Department of Pathology and Experimental Therapeutics, Faculty of Medicine and Health Sciences, Institute of Neurosciences, University of Barcelona, Av. Feixa Llarga s/n, 08907 L’Hospitalet de Llobregat, Spain; @ Neuropharmacology and Pain Group, Neuroscience Program, IDIBELL, Av. Feixa Llarga s/n, 08907 L’Hospitalet de Llobregat, Spain

## Abstract

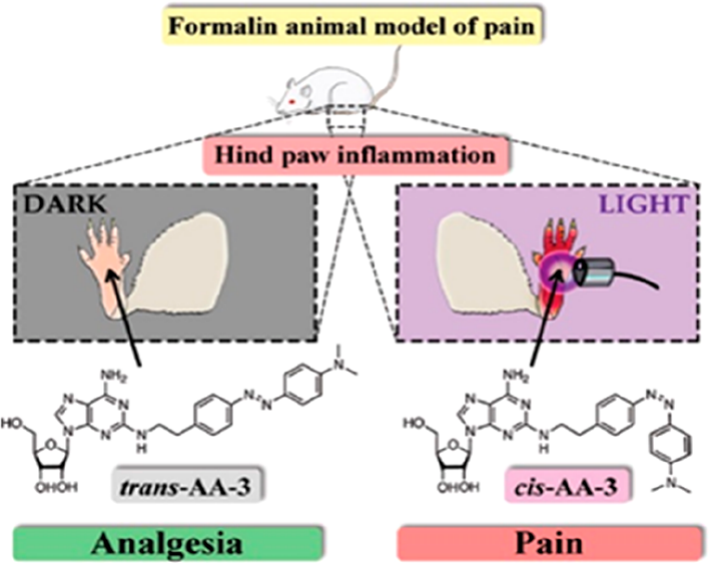

Adenosine receptors (ARs) play many important roles in physiology and have been recognized as potential targets for pain relief. Here, we introduce three photoswitchable adenosine derivatives that function as light-dependent agonists for ARs and confer optical control to these G protein-coupled receptors. One of our compounds, AzoAdenosine-3, was evaluated in the classical formalin model of pain. The molecule, active in the dark, was not metabolized by adenosine deaminase and effectively reduced pain perception in a light-dependent manner. These antinociceptive effects suggested a major role for A_1_R and A_3_R in peripheral-mediated pain sensitization, whereas an average adenosine-mediated antinociceptive effect will be facilitated by A_2A_R and A_2B_R. Our results demonstrate that a photoswitchable adenosine derivative can be used to map the contribution of ARs mediating analgesia *in vivo*.

Photopharmacology aims to provide optical control over biological function through the application of synthetic photoactivatable molecules to a broad range of endogenous or engineered receptors. Initially explored with ion channels and enzymes, most significant progress has occurred with G protein-coupled receptors (GPCRs),^1^ including adenosine receptors (ARs).^2,3^ This may be due to the fact that the only photoreceptors in the human genome, the opsins, are GPCRs with a covalently attached photoswitchable molecule (retinal) and that many GPCRs are well suited to accommodate a photoswitchable ligand and respond to its light-induced conformational changes. GPCRs are one of the most important protein classes for drug development.^4^ However, the roles of many receptors and their subtypes in health and disease are not yet fully understood.

A case in point are ARs, purinergic receptors that belong to the rhodopsin-like family of class A GPCRs.^5^ ARs are divided in four subtypes, A_1_, A_2A_, A_2B_, and A_3_. The role of adenosine in antinociception was initially identified in the 1970s and further investigated in the 1980s by systemic or intrathecal administration of selective ARs agonists in animal models of pain.^6^ While A_1_R was initially the major receptor of interest, a number of recent studies have focused on other AR subtypes.^7,8^ Thus, A_1_R and A_3_R have been clearly identified as potential targets for pain relief, while some controversy exists regarding the role (pro-nociceptive vs antinociceptive) of A_2A_R.^6,8^ Nevertheless, it seems clear that endogenous adenosine may contribute to the efficacy of pain-relieving mechanisms, thus adenosine derivatives have emerged as potential analgesics and antinociceptive agents.^9^

We describe a photoswitchable derivative of the endogenous agonist adenosine, which can be used to reversibly activate certain AR subtypes and to investigate the contributions of different AR subtypes in pain transmission.

Adenosine derivatives substituted in position 2 of the purine usually display selectivity for A_2A_R, A_2B_R, and A_3_R over A_1_R, where only small substituents are tolerated in this position.^10^ However, bulky substitutions in the *N*-6 position are mainly found in A_1_R selective agonists, but also in A_3_R selective agonists. We aimed to synthesize broadly applicable photoswitchable agonists and therefore decided to attach an azobenzene photoswitch in the 2-position of adenosine (Figure 1). We hypothesized that the two azobenzene isomers would interact differently with the extracellular loops of ARs, similar to what has been suggested for an *N*-6-substituted photoswitchable adenosine.^2^

**Figure 1:**
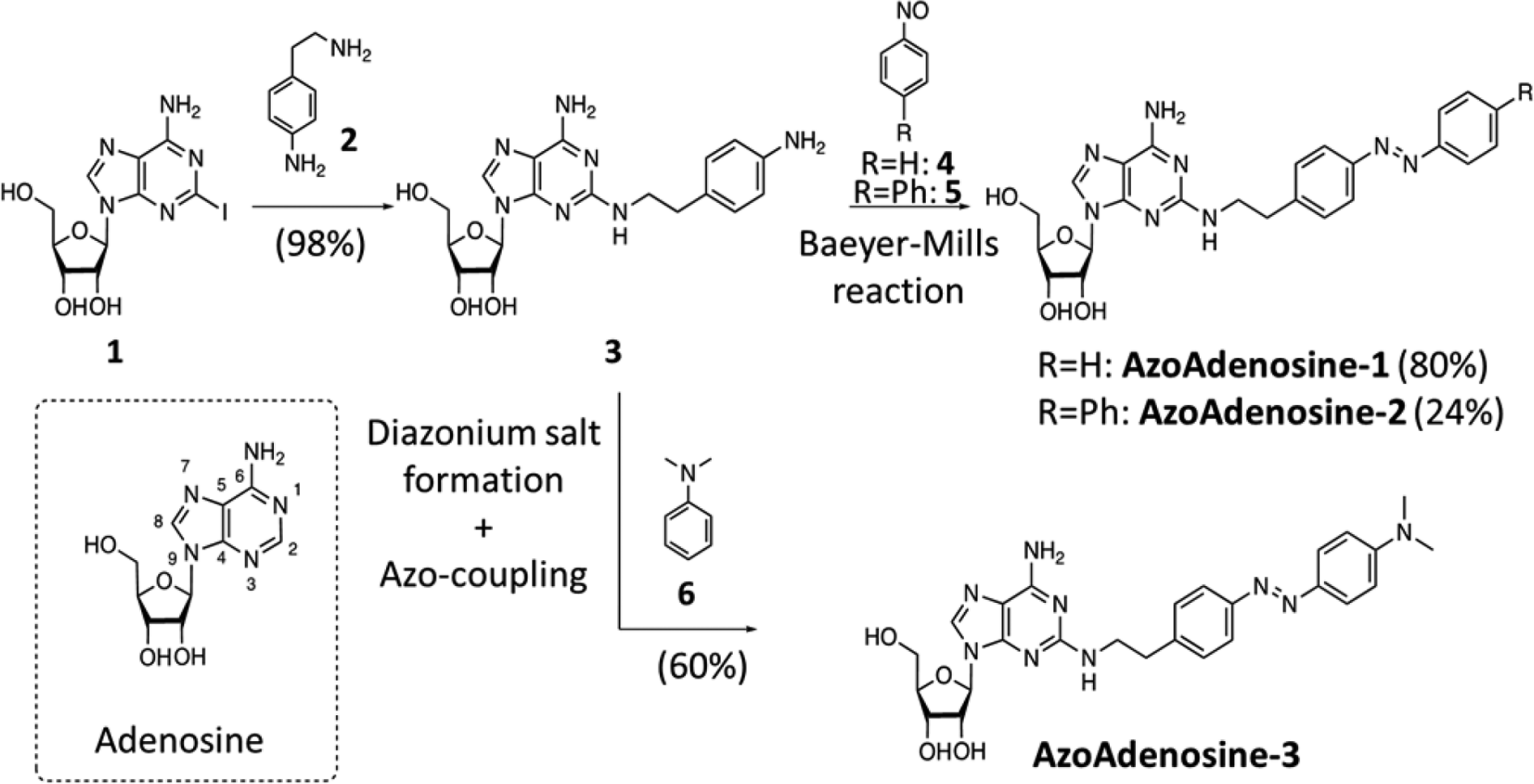
Synthesis of photoswitchable AR agonists and molecular structure of adenosine. Synthesis of **AA**-**1** and **AA-2** was achieved through azo-coupling, synthesis of **AA-3** by a Baeyer-Mills reaction (see Supporting Information for specific details of drug synthesis).

The synthesis of our photoswitchable AR ligands started from commercially available 2-iodoadenosine (**1**), which gave the common precursor **3** after an S_N_Ar with aminophenethylamine (**2**) (Figure 1).^11^ Compound **3** underwent chemoselective Baeyer-Mills reactions^12,13^ with nitrosoarenes **4** or **5**, without protection of the ribose moiety or aminopurine, to yield AzoAdenosine-1 (**AA-1**) and AzoAdenosine-2 (**AA-2**), respectively (see Supporting Information). The red-shifted photoswitchable adenosine derivative AzoAdenosine-3 (**AA-3**) was prepared via chemoselective diazotization of **3** and subsequent azo coupling with *N*,*N*-dimethyl aniline.^14^ The corresponding Baeyer-Mills reaction was unsuccessful in this case.

UV–vis spectrophotometry was employed to determine the optimal isomerization wavelengths and the isomerization properties of the photoswitchable AR ligands. Thus, while **AA-1** was most efficiently *cis* isomerized by irradiation with 360 nm illumination (Figure 2a), the **AA-2** could be *cis* isomerized with 380 nm light (Figure 2b). In both cases, a wide range of blue light (400–460 nm and 420–480 nm, respectively) could be used to facilitate *cis* to *trans* isomerization either in DMSO (Figure 2c and d) or in PBS (Figure 2e and f). The λ_max_ of **AA-3** showed a pronounced bathochromic shift of the absorption maximum to 420 nm, as well as overlapping π–π* and n−π* transitions (Figure 2g). As generally observed with red-shifted azobenzenes, we measured accelerated thermal dark-relaxation for **AA-3** (τ_off_ = 7.4 s; Figure 2h) compared to **AA-1** and **AA-2** (negligible relaxation over 30 min). Therefore, a significant change of the photostationary state (PSS) could only be observed upon irradiation in the nonprotic solvent DMSO (to slow down thermal relaxation, compared to protic solvents like PBS) and using higher-intensity light (390 and 460 nm ultra-high-power LEDs and 415 nm Mic-LED by Prizmatix). Experiments with **AA-3** in protic solvents did not result in an observable change in *trans*/*cis* ratio (not shown). Nevertheless, we assumed photochemical isomerization to occur, although it could not be observed with the employed UV–vis spectrophotometer.^15,16^ In contrast, thermostability of **AA-1** and **AA-2** was less affected by the solvent. **AA-1** and **AA-2** were *cis*-stable in the dark for at least 30 min, in both DMSO and PBS.

**Figure 2:**
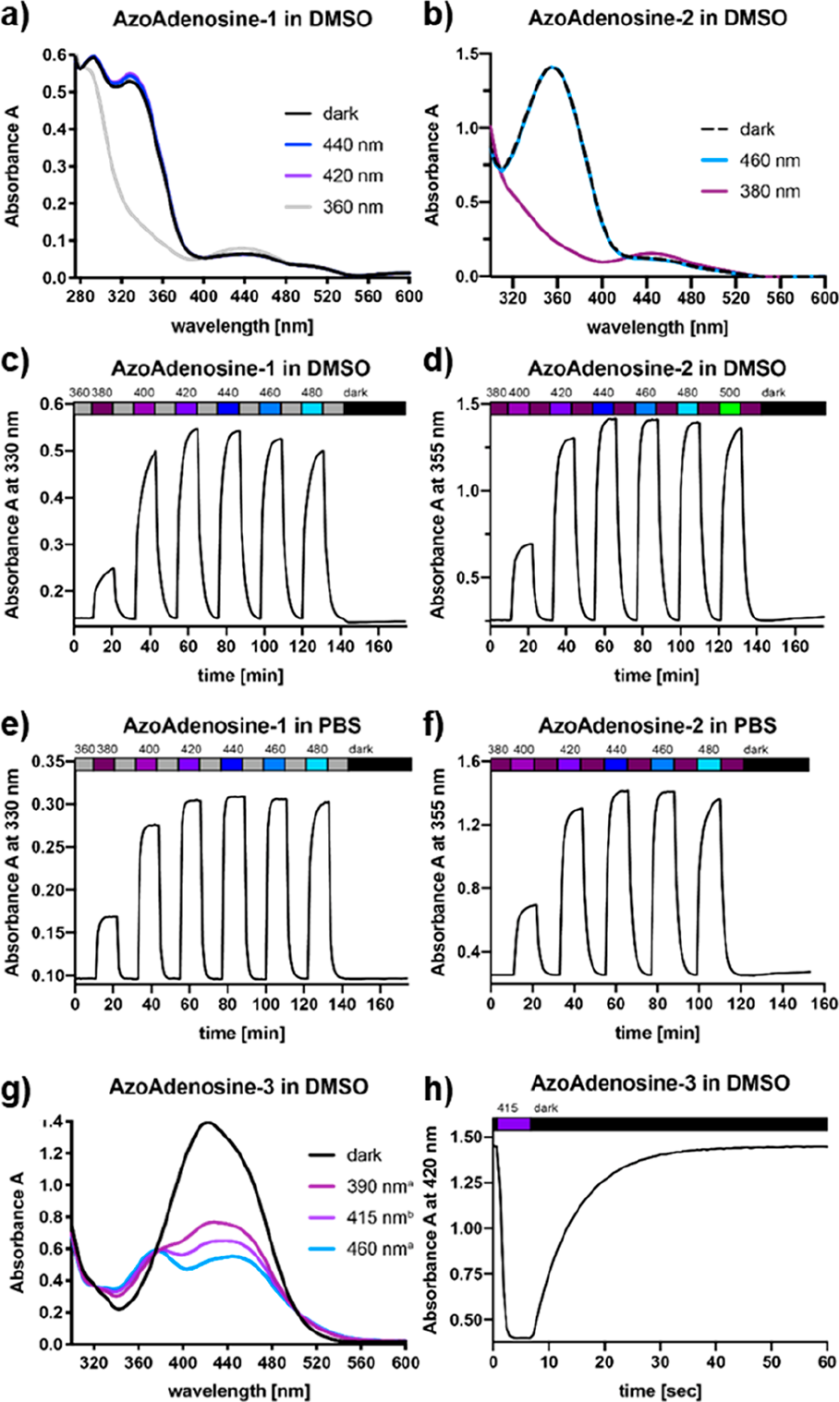
UV–vis studies of three AzoAdenosines in DMSO and/or PBS. Illumination-dependent UV–vis spectra of **AA-1** (a) and **AA-2** (b). Alternating illumination shows PSSs of **AA-1** (c–e) and **AA-2** (d–f) in DMSO and PBS. Illumination-dependent UV–vis spectra of **AA-3** (g) and its thermal relaxation (h) in DMSO (τ_on_ = 0.5 s, τ_off_ = 7.4 s). All spectra recorded at 50 μM and room temperature.

Additionally, PSS quantification in DMSO was achieved by NMR spectroscopy (see Figure S1, Supporting Information).^17^ Under ambient light conditions, a DMSO solution of **AA-1,2,3** contained 89%, 84%, and 87% *trans*-isomer. Illumination with the respective wavelengths (**AA-1**: 360 nm; **AA-2**: 380 nm; **AA-3**: 415 nm) enriched the *cis*-content to 85%, 68%, and 74% respectively (see Figure S1, Supporting Information). Isomerization to the thermodynamically favored isomer (**AA-1**: 420 nm; **AA-2**: 460 nm) was incomplete, with residual 28% and 16% *cis*-isomer.

Subsequently, radioligand binding studies were performed to determine AR selectivity for the photoswitchable ligands.^18^ The data revealed that **AA-1** and **AA-3** bind A_1_R, A_2A_R, and A_2B_R, with weak selectivity for A_2A_R over A_2B_R over A_1_R (see Table S1, Supporting Information). **AA-2** binds A_2A_R and A_2B_R, in agreement with our pharmacological design hypothesis. *K*_i_ values for all AR ligand binding were found to be in the nanomolar (**AA-1** and **AA-3**) and low micromolar (**AA-2**) range (see Table S2, Supporting Information).

As endogenous adenosine prevents hyperalgesia,^6^ we explored the antinociceptive profile of our photoswitchable adenosine derivatives in a preclinical model, the formalin-based hind paw inflammatory pain model. Out of the three azoadenosines developed, we selected **AA-3** to investigate light-dependent antinociceptive efficacy as its optimal photoconversion is achieved with visible spectrum light (415 nm), which it has better tissue penetration and causes less tissue damage than UV light. Mice received a subplantar injection of formalin in the hind paw, which leads to a characteristic biphasic nociceptive response: the initial phase (0–5 min), reflecting acute pain, and a second phase (15–30 min), due to central sensitization.^19^ Formalin injection in the hind paw induced an innate licking behavior, which was significantly reduced in both phases by a previous local administration of **AA-3** in dark conditions (Figure 3). The effect of **AA-3** was higher in phase II, which could be related to a major role of ARs to central sensitization.^6^ Interestingly, we could not observe an antinociceptive effect of adenosine itself under the same administration regime (i.e., intraplantar, 5 mM/50 nmol) (Figure 3), and when **AA-3** injection was followed by irradiation with 405 nm at the injection site, the antinociceptive effect of the compound was abolished, thus indicating that **AA-3** could be photomodulated *in vivo* (Figure 3).

**Figure 3:**
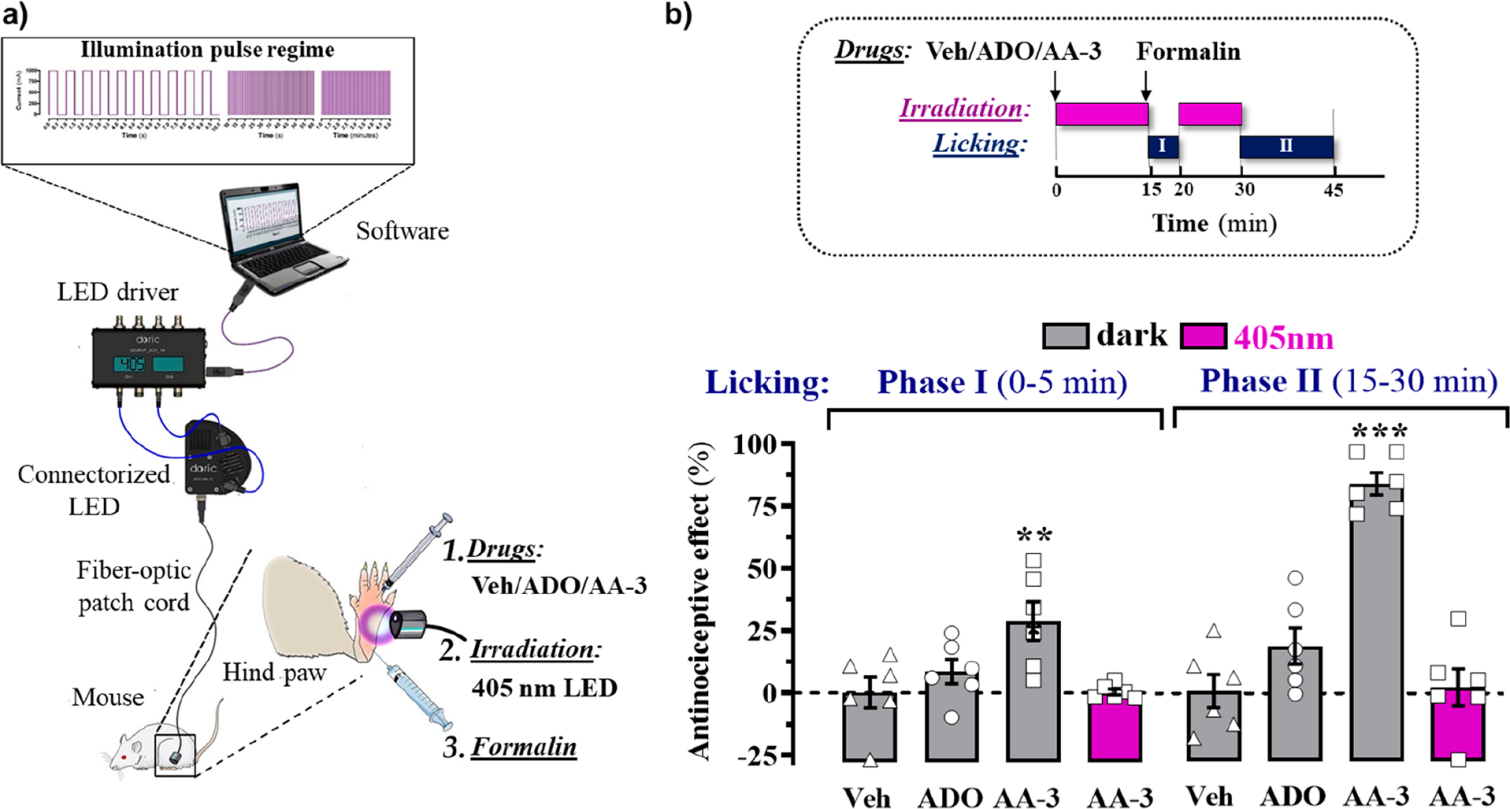
Light-dependent antinociceptive effect of AA-3 in mice. (a) Schematic representation of the experimental setup for *in vivo* photopharmacology. The different elements of the setup are depicted and the illumination regime and mouse hind paw manipulations indicated. (b) In the upper panel a scheme of the irradiation regime at 405 nm light (violet rectangles) and licking recordings (dark blue rectangles — Phase I and Phase II) in the formalin animal model of pain is shown. Mice were intraplantarly injected (10 μL) with vehicle (Veh, 20% DMSO + 20% Tween-80 in saline) Adenosine (ADO, 5 mM/50 nmols) or **AA-3** (5 mM/50 nmols) and irradiated with 405 nm light or mock-manipulated for 15 min. Subsequently, formalin was injected (20 μL, 2.5% formalin/0.92% formaldehyde), and the total hind paw licking measured during 15–20 min (Phase I) and 30–45 min (Phase II). The antinociceptive effect was calculated as the percentage of the maximum possible effect (mean ± S.E.M., *n* = 6 mice per group). **P < 0.01 and ***P < 0.001, one-way ANOVA with Dunnett’s multiple comparison test using Veh as a control.

These results support that, apart from allowing a spatial and temporal control of **AA-3**-mediated antinociceptive effects, the azobenzene group may protect **AA-3** from purine clearance systems (*i.e*., transport and metabolism). Indeed, adenosine deaminase (ADA) rapidly metabolizes adenosine from the extracellular milieu.^20^ Therefore, we evaluated **AA-3** sensitivity to ADA catalytic activity. To this end, ADA activity in the presence of adenosine and **AA-3** was determined *in vitro* by monitoring the reduction in absorbance at 265 nm resulting from the deamination of adenosine.^21^ Importantly, adenosine, but not **AA-3**, was degraded by ADA after 5 min of enzyme incubation (see Figure S2, Supporting Information).

On the other hand, we interrogated whether **AA-3** may have central effects in behaving animals. Accordingly, we compared the effects on locomotion of the systemic administration (i.p.) of adenosine and **AA-3**. While adenosine did not affect locomotor activity, systemic **AA-3** administration showed a significant reduction in locomotor activity, as sedation was observed (see Figure S3, Supporting Information). These results indicated that **AA-3** can cross the brain blood barrier (BBB) to activate central ARs.

We reason that **AA-3** could be a valuable tool to elucidate the mechanism of action of adenosine and the contribution of the different receptor subtypes within the pain neuraxis. Accordingly, we aimed at determining the contribution of the different AR subtypes to the local **AA-3** antinociceptive effects. Thus, before local **AA-3** administration, we systemically administered selective A_1_R, A_2A_R, A_2B_R, and A_3_R antagonists (PSB36, SCH442146, PSB603, and MRS1523, respectively)^22−25^ and measured their effects in the two different phases of the pain response. While PSB36, PSB603, and MRS1523 were unable to block **AA-3**-mediated antinociceptive effect in Phase I, SCH442146 was able to reduce **AA-3** induced antinociception (Figure 4), thus indicating a potential participation of A_2A_R in this phase of pain transmission. However, in Phase II all ARs antagonist were able to block either totally (i.e., PSB36 and MRS1523) or partially (i.e., SCH442146 and PSB603) the **AA-3**-mediated antinociceptive effect (Figure 4), thus suggesting a differential participation of ARs in this phase of pain transmission. Based on these results, it appears that, in Phase I, **AA-3** might selectively act at A_2A_R. Conversely, the most potent **AA-3** antinociceptive effect in Phase II would be mainly mediated by interacting with A_1_R and A_3_R, whereas an average antinociceptive effect will be facilitated by A_2A_R and A_2B_R (Figure 4). These result are consistent with previous studies, wherein selective agonists for A_1_R and A_3_R (and with some controversy A_2A_R) showed antinociceptive efficacy (for review, see refs (6,26)).

**Figure 4:**
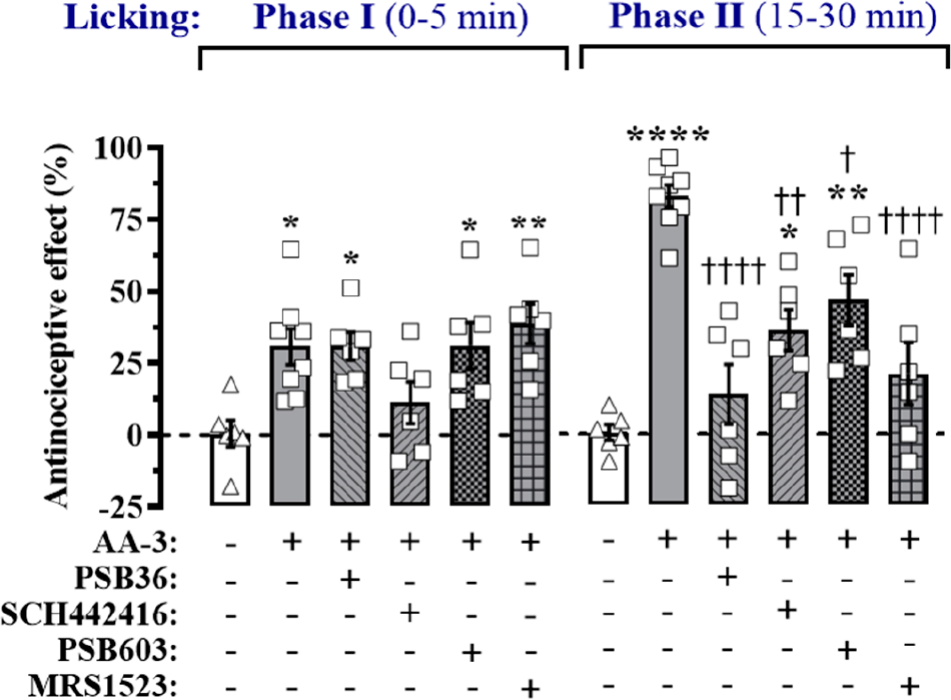
Mice were first intraperitoneally injected with vehicle (Veh, saline), PSB36 (3 mg/kg),^22^ SCH442146 (1 mg/kg),^23^ PSB603 (5 mg/kg),^24^ or MRS1523 (2 mg/kg).^25^ After 15 min, mice were locally injected (10 μL) with vehicle (20% DMSO + 20% Tween-80 in saline) or **AA-3** (5 mM/50 nmol), and 15 min later, animals received the formalin injection (20 μL, 2.5% formalin/0.92% formaldehyde). The antinociceptive effect was determined (see Figure 3) and expressed as a percentage of the maximum possible effect (mean ± S.E.M., *n* = 6–8 mice per group). *P < 0.05, **P < 0.01, and ****P < 0.0001, one-way ANOVA followed by Tukey’s posthoc test compared with cells treated with vwhicle; ^†^*P* < 0.05, ^††^*P* < 0.01, and ^††††^*P* < 0.0001, when compared with cells treated only with **AA-3**.

Our results suggest that peripheral ARs are responsible for central sensitization occurring at phase II of the pain response. Concretely, A_1_R and A_3_R, and with less extent A_2A_R and A_2B_R, would play a major role in such pain mechanisms, and the inhibition of pro-inflammatory and pro-nociceptive mediators from immune cells could be the main mechanism.^6,26^ Finally, it is important to note that it cannot be entirely ruled out that some distribution of **AA-3** after its intraplantar injection occurred. However, the significant dilution and the complete abrogation of **AA-3**-mediated antinociceptive effect upon local hind paw irradiation makes it unlikely that **AA-3** acts centrally when locally injected. Overall, our results may be viewed as a proof of concept, which consist of using a photoswitchable endogenous adenosine molecule to probe the contribution of ARs within the organism mediating anti-hyperalgesia.

## Supporting Information Available

Drug Synthesis, general materials and methods, biological data, analytical data (PDF)
