# Supplementary material for: Optical Control of Adenosine-Mediated Pain Modulation
Source: Bioconjug Chem. 2021 Aug 27;32(9):1979–83. doi: 10.1021/acs.bioconjchem.1c00387 (PMC8634359; doi:10.1021/acs.bioconjchem.1c00387)
Supplement: Supplementary file 1 — bc1c00387_si_001.pdf [file bc1c00387_si_001.pdf]

Supporting Information

Optical Control of Adenosine-Mediated Pain Modulation

Katharina Hüll[1,2#], Víctor Fernández-Dueñas[3,4#], Matthias Schönberger[2], Marc López-Cano[3,4], Dirk Trauner[1]*, and Francisco Ciruela[3,4]*

[1]Department of Chemistry, New York University, 100 Washington Square East, New York City, NY 10003, USA.
[2]Department of Chemistry and Center for Integrated Protein Munich, Ludwig-Maximilians-Universität Menchen, Butenandtstrasse 5–13, 81377 Munich, Germany.
[3]Pharmacology Unit, Department of Pathology and Experimental Therapeutics, Faculty of Medicine and Health Sciences, Institute of Neurosciences, University of Barcelona, Av. Feixa Llarga s/n, 08907 L'Hospitalet de Llobregat, Spain.
[4]Neuropharmacology and Pain Group, Neuroscience Program, IDIBELL, Av. Feixa Llarga s/n, 08907 L'Hospitalet de Llobregat, Spain.

[#]Contributed equally to this work
*Corresponding authors

Table of Contents

Drug Synthesis	S3
Methods	S9
Analytical Data	S12
NMR Spectra	S12
Figure S1. Determination of photostationary states (PSSs)	S16
Biological Data	S17
Figure S2. Time course of adenosine deaminase activity	S17
Figure S3. AA-3 induced locomotor activity	S18
Table S1: Inhibition of binding of hot ligand to ARs	S19
Table S2: Binding constants of AA-1, AA-2 and AA-3	S20
References	S21

Drug Synthesis

Unless stated otherwise indicated all reactions were carried out with magnetic stirring using oven-dried glassware (160°C) under inert gas atmosphere (nitrogen or argon). Syringes used to transfer reagents and solvents were purged with nitrogen prior to use. Low temperature reactions were carried out in a Dewar vessel filled with the appropriate cooling agent e.g. H_2O/ice (0°C). Heating was conducted using a heated oil bath. Yields refer to spectroscopically pure compounds unless otherwise stated.

Reaction solvents were purchased from Acros Organics as 'extra dry' over molecular sieves and handled under inert gas atmosphere. Tetrahydrofuran (THF) was distilled from Na/benzophenone prior to use. Dichloromethane (DCM), triethylamine (TEA) and diisopropylethylamine (DIPEA) were distilled from calcium hydride. Ethanol and acetic acid were purchased from commercial suppliers and used as received. Solvents for extraction and flash column chromatography were purchased in technical grade purity and distilled under reduced pressure on a rotary evaporator prior to use. All other reagents and solvents were purchased from commercial suppliers and used as received.

Reactions and chromatography fractions were monitored by qualitative thin-layer chromatography (TLC) on silica gel F254 TLC plates from Merck KGaA. Analytes were visualized by irradiation with UV light and/or by immersion of the TLC plate in ninhydrin or potassium permanganate solution followed by heating with a hot-air gun. Flash column chromatography was performed Geduran® Si60 (4063 μm) silica gel from Merck KGaA (eluents are given in parentheses). Reverse Phase column chromatography was performed on Waters C18 (C18; 55-105 μm, 125 Å) as stationary phase (eluents are given in parentheses). LCMS was performed on an Agilent 1260 Infinity HPLC System, MS-Agilent 1100 Series, Type: 1946D, Model: SL, equipped with an Agilent Zorbax Eclipse Plus C18 (100 x 4.6 mm,

particle size 3.5 micron) reverse phase column with a constant flowrate of 1 mL/min and a 10 → 100% MeCN/H2O + 0.1% FA gradient over 10 min.

NMR spectra were measured on Varian 400 MHz Bruker AVIII HD (cryoprobe) for proton nuclei (100 MHz for carbon nuclei respectively). The 1H NMR shifts are reported in parts per million (ppm) related to the chemical shift of tetramethylsilane. 1H and 13C NMR shifts were calibrated to the residual solvent signal: DMSO (2.50 ppm/39.5 ppm). 1H NMR spectroscopic data are reported as follows: Chemical shift in ppm (multiplicity, coupling constants (Hz), integration). The multiplicities are abbreviated as follows: s (singlet), d (doublet), t (triplet), q (quartet) and m (multiplet) and are reported as observed. Except for multiplets, the chemical shift of all signals is reported as the center of the resonance range. Additionally, to 1H and 13C NMR measurements, 2D NMR techniques as homonuclear correlation spectroscopy (COSY), heteronuclear single quantum coherence (HSQC) and heteronuclear multiple bond coherence (HMBC) were used to assist signal assignment. All raw fid files were processed, and the spectra analyzed using the program MestReNova 11.0 from Mestrelab Research S. L.

All high-resolution mass spectra (HRMS) were recorded by the LMU Mass Spectrometry Service. HRMS were recorded on a MAT 90 from Thermo Finnigan GmbH using electrospray ionisation (ESI) or a MAT 90 from Jeol Ltd. using electron ionization (EI).

UV/Vis spectra were recorded on a Varian Cary 50 Scan UV/Vis spectrometer using Helma SUPRASIL precision cuvettes (10 mm light path). All compound stock solutions were prepared under benchtop light conditions at 50 mM in DMSO and diluted to 50 μM in the final solvent (DMSO or PBS). Photoswitching was achieved using a Polychrome V (Till Photonics) monochromator, or a Prizmatix ultra high-power LED (460 nm), connected to a fiber-optic cable through which the sample in the spectrophotometer was irradiated from the top.

The synthesis of (2*R*,3*R*,4*S*,5*R*)-2-(6-amino-2-((4-aminophenethyl)amino)-9H-purin-9-yl)-5-(hydroxymethyl)tetrahydrofuran-3,4-diol (3), AzoAdenosine-1, AzoAdenosine-2 and AzoAdenosine-3 has been reported elsewhere[1,2] and is summarized below.

(2*R*,3*R*,4*S*,5*R*)-2-(6-amino-2-((4-aminophenethyl)amino)-9H-purin-9-yl)-5-(hydroxymethyl)tetrahydrofuran-3,4-diol (3)

2-Iodoadenosine (**1**) (150 mg, 0.382 mmol, 1.0 eq.) was suspended in neat amino phenethylamine (**2**) (251 µL, 1.91 mmol, 5.0 eq) and heated to 110°C under Ar atmosphere. After turning homogenous at approximately 70°C, the mixture was stirred at 110°C for 3 h. The reaction mixture was allowed to cool to rt, dissolved in methanol and purified using silica gel column chromatography (10% MeOH/DCM). **3** was obtained as an off-white foam (150 mg, 0.374 mmol, 98%).

^{1}H NMR (400 MHz, DMSO-*d*$_6$): δ = 7.90 (s, 1H), 6.90 (d, *J* = 8.1 Hz, 2H), 6.73 (s, 2H), 6.49 (d, *J* = 8.1 Hz, 2H), 6.03 (t, *J* = 5.4 Hz, 1H), 5.73 (d, *J* = 6.0 Hz, 1H), 5.37 (d, *J* = 6.1 Hz, 1H), 5.13 (s, 1H), 5.10 (d, *J* = 4.6 Hz, 1H), 4.82 (s, 2H), 4.62 (s, 1H), 4.17 – 4.06 (m, 2H), 3.89 (d, *J* = 3.5 Hz, 1H), 3.68 – 3.59 (m, 1H), 3.58 – 3.50 (m, 1H), 2.64 (t, *J* = 7.3 Hz, 2H).

^{13}C NMR (100 MHz, DMSO-*d*$_6$): δ = 159.2, 156.0, 151.5, 146.6, 136.4, 129.1, 126.9, 114.0, 113.6, 87.2, 85.3, 72.9, 70.7, 61.8, 43.4, 34.7.

Note: ^{1}H and ^{13}C show signals of residual MeOH.

HRMS (ESI) calcd for $C_{18}H_{24}N_7O_4$ $[M+H]^+$: 402.1884; found: 402.1881

R$_f$ = 0.2 (10% MeOH/DCM)

AzoAdenosine-1 (AA-1)

3 (60 mg, 0.149 mmol, 1.0 eq.) and nitrosobenzene (**4**) (40.0 mg, 0.347 mmol, 2.5 eq.) were dissolved in DCM (2 mL) and acetic acid (200 µL, 0.349 mmol, 23.0 eq.) was added. The

reaction mixture was stirred overnight and neutralized with $NaHCO_3$. The phases were separated, and the organic phase was washed with $NaHCO_3$, the aqueous phase was extracted with EtOAc (3 x) and the combined organic layers were dried over Na_2SO_4 and concentrated *in vacuo*. The crude material was purified by silica gel column chromatography (10% MeOH/DCM) to yield **AA-1** (58.0 mg, 0.118 mmol, 80%) as orange solid.

^{1}H NMR (400 MHz, DMSO-*d*$_6$): δ = 7.92 (s, 1H), 7.86 (dd, *J* = 15.5, 8.0 Hz, 4H), 7.59 (q, *J* = 9.6, 8.6 Hz, 3H), 7.50 (d, *J* = 8.0 Hz, 2H), 6.77 (s, 2H), 6.31 (s, 1H), 5.76 (d, *J* = 5.9 Hz, 1H), 5.39 (d, *J* = 5.8 Hz, 1H), 5.13 (d, *J* = 4.1 Hz, 2H), 4.64 (s, 1H), 4.15 (s, 1H), 3.91 (d, *J* = 3.4 Hz, 1H), 3.69 – 3.59 (m, 1H), 3.59 – 3.46 (m, 3H), 2.95 (t, *J* = 7.1 Hz, 2H).

^{13}C NMR (100 MHz, DMSO-*d*$_6$): δ = 159.7, 156.5, 152.4, 151.9, 150.8, 144.9, 136.9, 131.8, 130.3, 129.9, 123.1, 122.9, 114.2, 87.6, 85.7, 73.4, 71.1, 62.3, 43.1, 35.7.

HRMS (ESI) calcd for $C_{24}H_{26}N_8O_4$ $[M+H]^+$: 491.2150; found: 491.2147.

R_f = 0.7 (20% MeOH/DCM).

λ_{max} (DMSO): 292 nm, 328 nm ($\pi \rightarrow \pi^*$).

AzoAdenosine-2 (AA-2)

Aminobiphenyl (189 mg, 1.12 mmol, 4.5 eq.) was dissolved in DCM (5 mL) and H_2O (10 mL). Oxone (689 mg, 1.12 mmol, 4.5 eq.) in H_2O (5 mL) was slowly added to the solution. After vigorous stirring overnight, the reaction mixture was diluted with EtOAc. The organic layer was washed with HCl (1 M), NaOH (1 M) and sat. NaCl, and dried over Na_2SO_4. After removal of the solvent under reduced pressure, the crude nitrosoarene was used without further purification as a DCM solution. **3** (100 mg, 0.249 mmol, 1.0 eq.) and AcOH (75.0 mg, 1.25 mmol, 71.0 µL, 5.0 eq.) were added to the solution and stirred for 23 h. The reaction mixture was neutralized washed with NaOH (1 M, 2 x), sat. NaCl and dried over Na_2SO_4. Purification using silica gel flash column chromatography (SiO_2, 5% → 50 % MeOH/DCM), followed by

reverse phase column chromatography (40 % MeCN/H_2O), yielded **AA-2** (34.0 mg, 60.1 μmol, 24 %) as brown solid.

^{1}H NMR (400 MHz, DMSO-*d*$_6$): δ = 7.98 (d, *J* = 8.7 Hz, 2H), 7.93 (s, 1H), 7.90 (d, *J* = 8.7 Hz, 2H), 7.86 (d, *J* = 8.4 Hz, 2H), 7.78 (d, *J* = 7.2 Hz, 2H), 7.51 (t, *J* = 7.6 Hz, 4H), 7.46 – 7.40 (m, 1H), 6.77 (s, 2H), 6.31 (t, *J* = 5.6 Hz, 1H), 5.77 (d, *J* = 6.0 Hz, 1H), 5.21 (s, 2H), 4.65 (s, 1H), 4.19 – 4.14 (m, 1H), 3.92 (q, *J* = 3.9 Hz, 1H), 3.66 (dd, *J* = 11.9, 4.0 Hz, 1H), 3.60 – 3.48 (m, 3H), 2.96 (t, *J* = 7.1 Hz, 2H).

^{13}C NMR (100 MHz, DMSO-*d*$_6$): δ =159.2, 156.1, 151.5, 151.1, 150.5, 144.4, 142.8, 139.0, 129.1, 128.2, 127.7, 126.9, 123.2, 122.7, 113.7, 87.2, 85.3, 73.0, 70.7, 61.8, 42.7, 35.3.

HRMS: (ESI) calcd. for $C_{30}H_{30}N_8O_4$ $[M+H]^+$: 567.2390; found: 567.2459.

R_f = 0.4 (10 % MeOH/DCM).

λ_{max} (DMSO): 355 nm ($\pi \rightarrow \pi^*$).

AzoAdenosine-3 (AA-3)

3 (100 mg, 0.249 mmol, 1.0 eq) was dissolved in methanol/water (50 mL, 1:1), cooled to 0 C and aq. HCl (1M, 747 μL, 0.747 mmol, 3.0 eq) was added. $NaNO_2$ was dissolved in a water (0.5 mL) and added dropwise to the stirred aniline solution. The reaction mixture was stirred for 30 min and the resulting solution of diazonium salt was added dropwise to a pre-cooled (0°C) mixture of dimethylaniline (**6**) (47.7 μL, 45.3 mg, 0.374 mmol, 1.5 eq.) and sodium acetate (123 mg, 1.49 mmol, 6.0 eq.) and in methanol. The resulting solution was slowly allowed to warm up to room temperature and stirred for 3 h. Ethyl acetate (110 mL) was added and the majority of the methanol was removed under reduced pressure. The organic phase was washed with sat. $NaHCO_3$ and sat. NaCl and dried over $MgSO_4$. The solvent was removed *in vacuo* and the crude material was purified by silica gel column chromatography (10 % MeOH/DCM) and **AA-3** (80.0 mg, 0.249 mmol, 60 %) was isolated as orange solid.

^{1}H NMR (400 MHz, DMSO-d_6): δ = 7.90 (s, 1H, CH), 7.75 (d, J = 8.7 Hz, 2H, CH_2), 7.69 (d, J = 7.9 Hz, 2H, CH2), 7.38 (d, J = 8.0 Hz, 2H, CH_2), 6.85 – 6.69 (m, 4H, NH_2 and CH_2), 6.28 (s, 1H, OH), 5.73 (d, J = 5.9 Hz, 1H, CH), 5.38 (d, J = 6.1 Hz, 1H, OH), 5.12 (d, J = 4.6 Hz, 2H, OH, overlapping with 1H, NH), 4.61 (s, 1H, CH), 4.16 – 4.08 (m, 1H, CH), 3.88 (d, J = 3.4 Hz, 1H, CH), 3.61 (dt, J = 8.7, 4.3 Hz, 1H, ½ CH_2), 3.55 – 3.42 (m, 3H, ½ CH_2 and CH_2), 3.02 (s, 6H, 2 CH_3), 2.88 (t, J = 7.1 Hz, 2H, CH_2).

^{13}C NMR (100 MHz, DMSO-d_6): δ = 159.2, 156.1, 152.4, 151.5, 150.8, 142.6, 142.2, 136.5, 129.6, 124.6, 121.9, 113.7, 111.6, 87.2, 85.3, 72.9, 70.7, 61.8, 42.8, 39.7 (extracted from HSQC), 35.2.

HRMS (ESI): calcd. for $C_{26}H_{32}N_9O_4$ $[M+H]^+$: 534,2572; found: 534.2572.

R_t: 2.866 min (5 → 100% MeCN/H_2O + 0.1% FA)

λ_{max} (DMSO): 422 nm (π → π*).

Methods

Binding Studies. Determination of binding inhibition and Ki values has been conducted by the psychoactive drug screen program[3] and a link to their protocol can be found on the program's website: https://pdsp.unc.edu/pdspweb/

Determination of trans/cis-ratios. NMR samples of 5 mM concentration were prepared, wrapped in tin foil and a sand-blasted glass fiber was adjusted to the correct height of 1 cm above the bottom of the NMR tube in the sample solution. The sample was inserted in a 200 MHz NMR machine and a test-spectrum was measured to ensure no interfering with the measurement. 1H NMRs were collected, every 5-10 min (in the first hour) to every hour (for 12-14 h). For **AA-1** and **AA-2**, illumination was then switched on as soon as the collection of the first 1H-spectrum was finished and continued over the time course of the experiment. For the red-shifted derivative, an illumination inside the NMR was not practical. The sample was irradiated outside the NMR and the maximum *cis*-content of the PSS was extrapolated. For the collection of the dark-relaxation data, illumination was switched off and the sample was left in the NMR machine and spectra recorded every hour for 0.5-14h. Corresponding *trans* and *cis* signals were integrated, and the percentage was calculated.

Adenosine deaminase activity. Adenosine deaminase (ADA, EC 3.5.4.4) activity was determined at 25°C with 0.1 mM adenosine as reference substrate in 50 mM Tris-HCl buffer, pH 7.4, as previously described.[4] The decrease in the absorbance at 265 nm ($\Delta\varepsilon$ = 7800 M^{-1} cm^{-1}) resulting from the deamination of adenosine was monitored in an POLARstar plate-reader (BMG Labtech, Durham, NC, USA) using Greiner UV-Star® 96-well plates (Greiner Bio-one España, San Sebastián de los Reyes, Spain). Steady-state kinetic measurements were

performed in 50 mM Tris-HCl buffer (pH 7.4) using 0.1 mM of adenosine and azo-adenosine-3 and a constant enzyme concentration (0.02 U/ml).

Animals. Adult male and female CD-1 mice (animal facility of University of Barcelona) weighing 20–25 g were used. The University of Barcelona Committee on Animal Use and Care approved the protocol (number: 10034). Following the approved experimental protocol all animals were supervised daily to assess signs of adverse effects during treatment. A retrospective analysis of the protocol demonstrated that no corrective measures (i.e. use of analgesics) were needed. Animals were housed and tested in compliance with the guidelines provided by the Guide for the Care and Use of Laboratory Animals[5] and following the European Union directives (2010/63/EU). Mice were housed in groups of five in standard cages with *ad libitum* access to food and water and maintained under a non-reversed 12 h dark/light cycle (starting light period at 7:30 AM), 22°C temperature, and 66% humidity (standard conditions). All animal experimentation was carried out in a period comprehended between 9:00 AM to 6:00 PM by a researcher blind to drug treatments.

Open field. The open field apparatus consists of a 38 x 38 x 38 cm chamber, where mice were placed in a corner facing the wall and their horitzontal locomotor activity was recorded with a camera during 10 min, as previously described.[6]

Formalin animal model of pain. The formalin animal model of pain was performed as previously described[7]. In brief, mice (n = 6-8 per group) were administered intraplantary (i.pl.) with 10 µL of vehicle (20% DMSO + 20% Tween-80 in saline), ADO (5 mM/50 nmols) or AA-3 (5 mM/50 nmols) 15 min before a diluted formalin solution (20 µL of 2.5% formalin/0.92% formaldehyde; Sigma-Aldrich) was intraplantarly (i.pl.) injected in the mid-

plantar surface of the right hind paw of the mouse, as previously described.[8] The formalin-induced nociceptive behaviour in light or mock manipulated (dark) conditions was quantified as the time spent licking or biting the injected paw during the 30 min after the injection of formalin. The initial acute phase (0–5 min; phase I), reflecting the acute peripheral pain, was followed by a relatively short quiescent period (10 min), which was then followed by a prolonged response (15–30 min; phase II), which is related to the development of central nociceptive sensitization. For the peripheral photo-control of **AA-3**, the hind paw was directly irradiated with a LED-based fiber-optic system (Doric Lenses Inc.) at 405 nm light (or dark) before the recording of each phase (15 min in phase I and 10 min in phase II). The present interval was selected according to previous experiments, which showed non-optimal photo-control reliable processes for periods lessen than 15 min (data not shown). The 405 nm light pulses lasted 500 ms each and were administered at 1 Hz frequency with 23 mW output power and 2000 mA intensity. Antinociception induced by the different treatments was calculated with the equation:

$$\text{Antinociceptive effect (\%)} = [(\text{LTV} - \text{LTD}) / \text{LTV}] \times 100$$

where LTV and LTD represent the licking/biting time in the vehicle- and drug-treated animals, respectively.

Statistics. Data are represented as mean ± standard error of mean (SEM). The number of samples/animals (n) in each experimental condition is indicated in the corresponding figure legend. Data normality was assessed by the Shapiro-Wilk normality test. Outliers were assessed by the Grubbs' test; no animals were excluded. Comparisons among experimental groups were performed by Student's *t* test, one-way ANOVA with Dunnett's *post-hoc* test or by two-way ANOVA followed by Bonferroni *post-hoc* test using GraphPad Prism 6.01 (San Diego, CA, USA), as indicated. Statistical difference was accepted when $P < 0.05$.

Analytical data

NMR Spectra

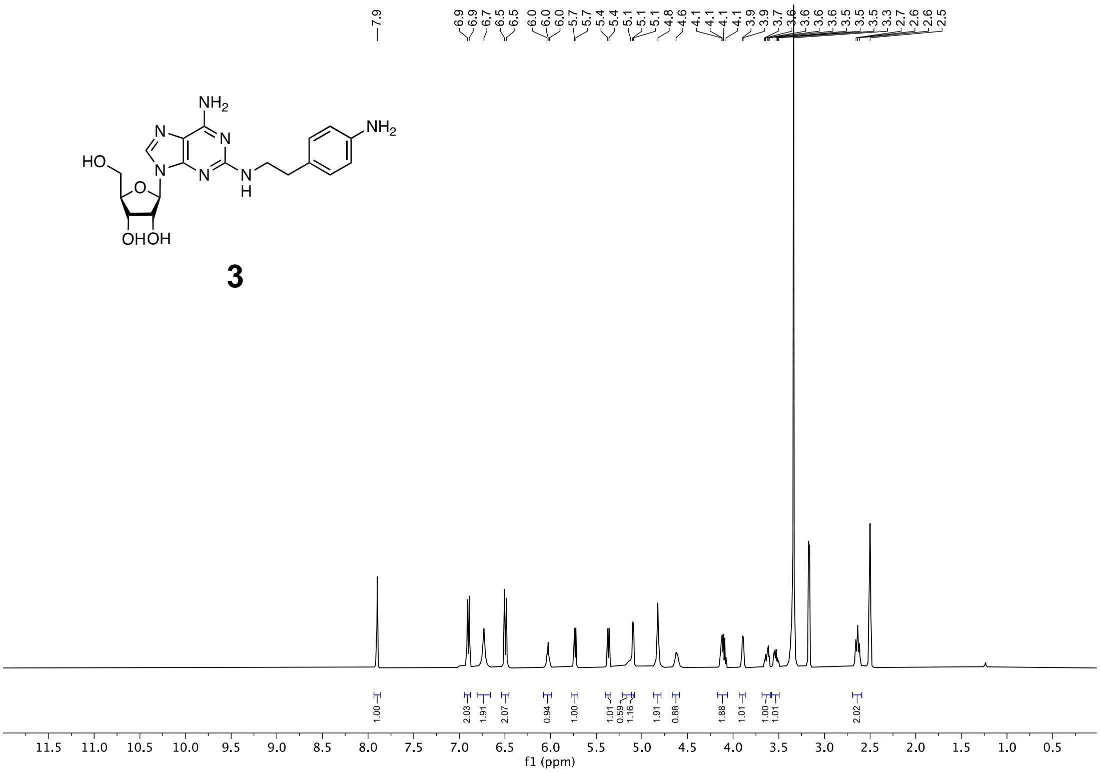

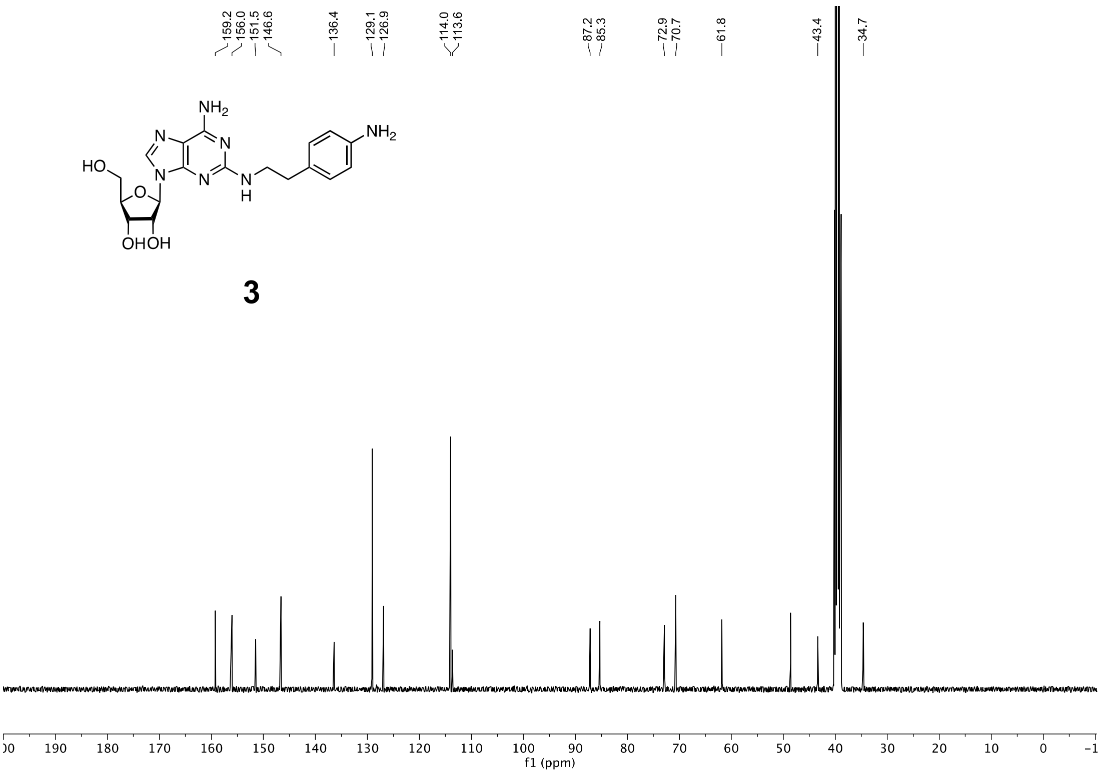

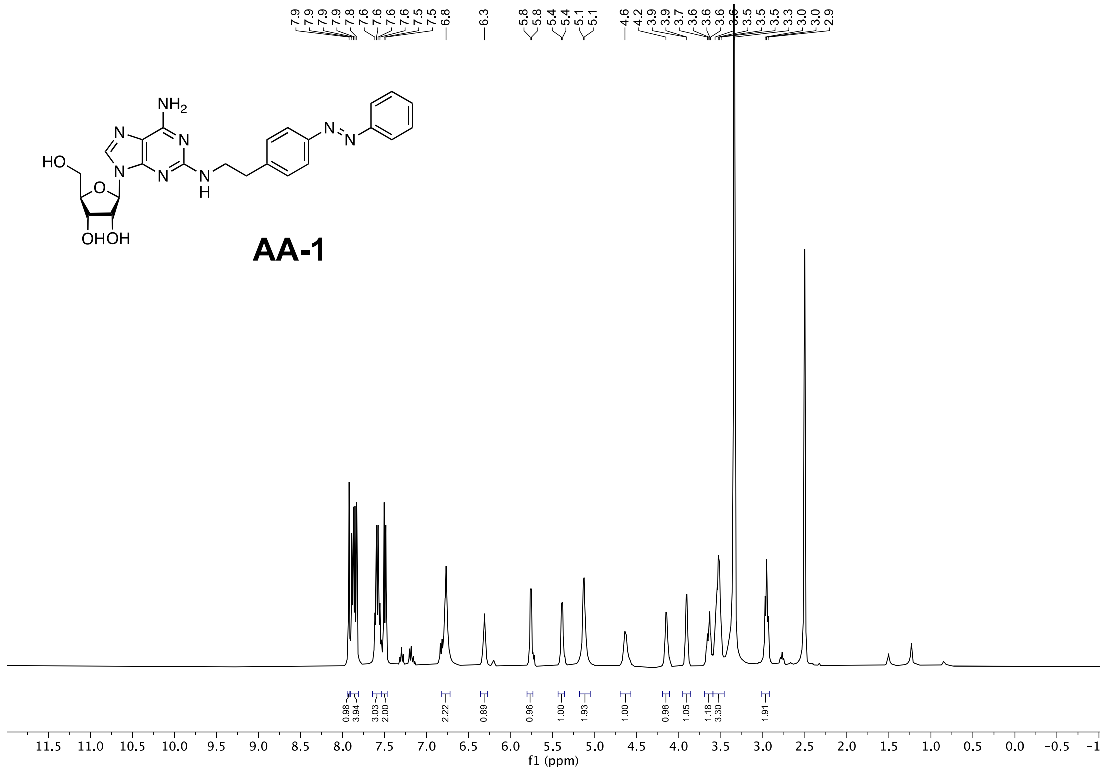
AA-1
f1 (ppm)

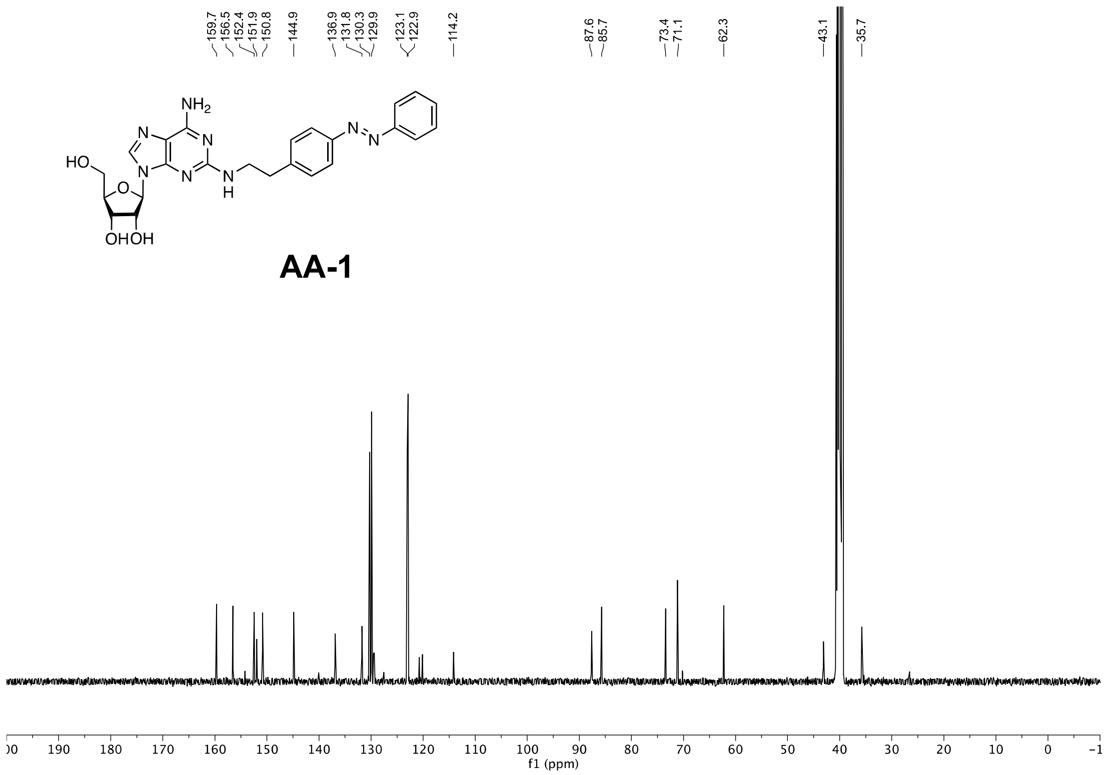
159.7
156.5
152.4
151.9
150.8
144.9
136.9
131.8
130.3
129.9
123.1
122.9
114.2
87.6
85.7
73.4
71.1
62.3
43.1
35.7
AA-1
f1 (ppm)

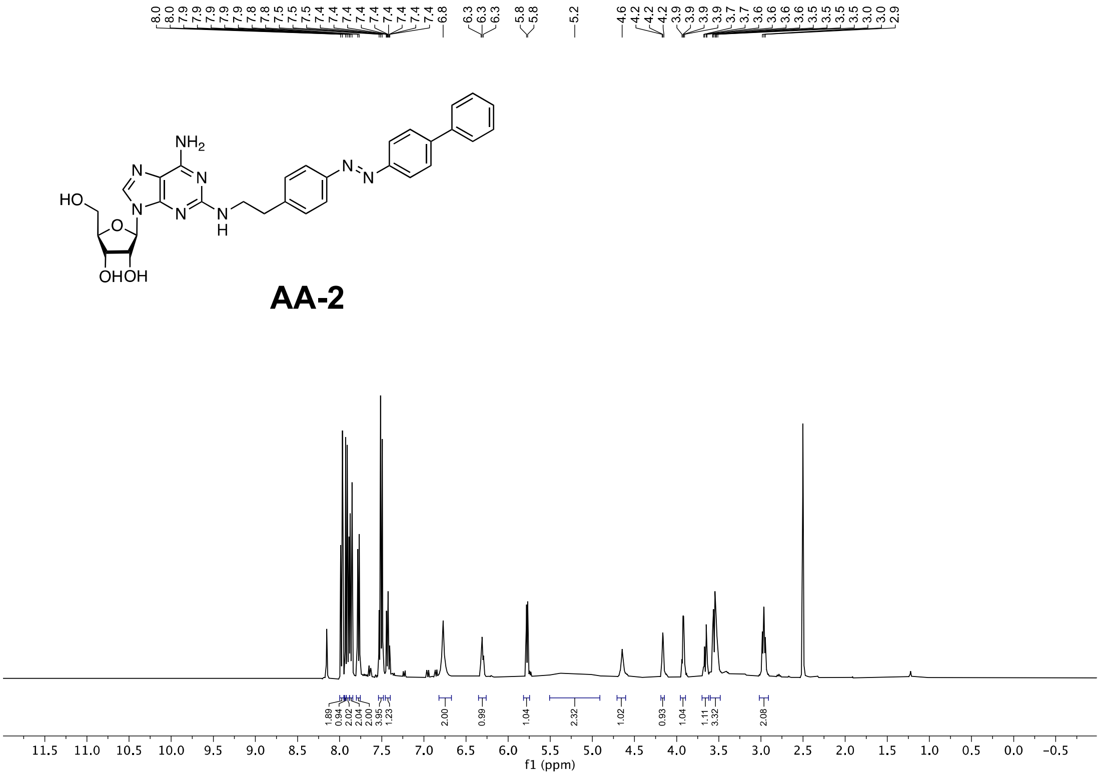
8.0 8.0 7.9 7.9 7.9 7.9 7.9 7.8 7.8 7.5 7.5 7.5 7.4 7.4 7.4 7.4 7.4 7.4 7.4 7.4 6.8 6.3 6.3 6.3 5.8 5.8 5.2 4.6 4.2 4.2 4.2 3.9 3.9 3.9 3.9 3.7 3.6 3.6 3.6 3.5 3.5 3.5 3.5 3.0 3.0 2.9
NH2
HO
OHOH
AA-2
1.89 0.94 2.02 2.04 2.00 3.95 1.23 2.00 0.99 1.04 2.32 1.02 0.93 1.04 1.11 3.32 2.08
f1 (ppm)

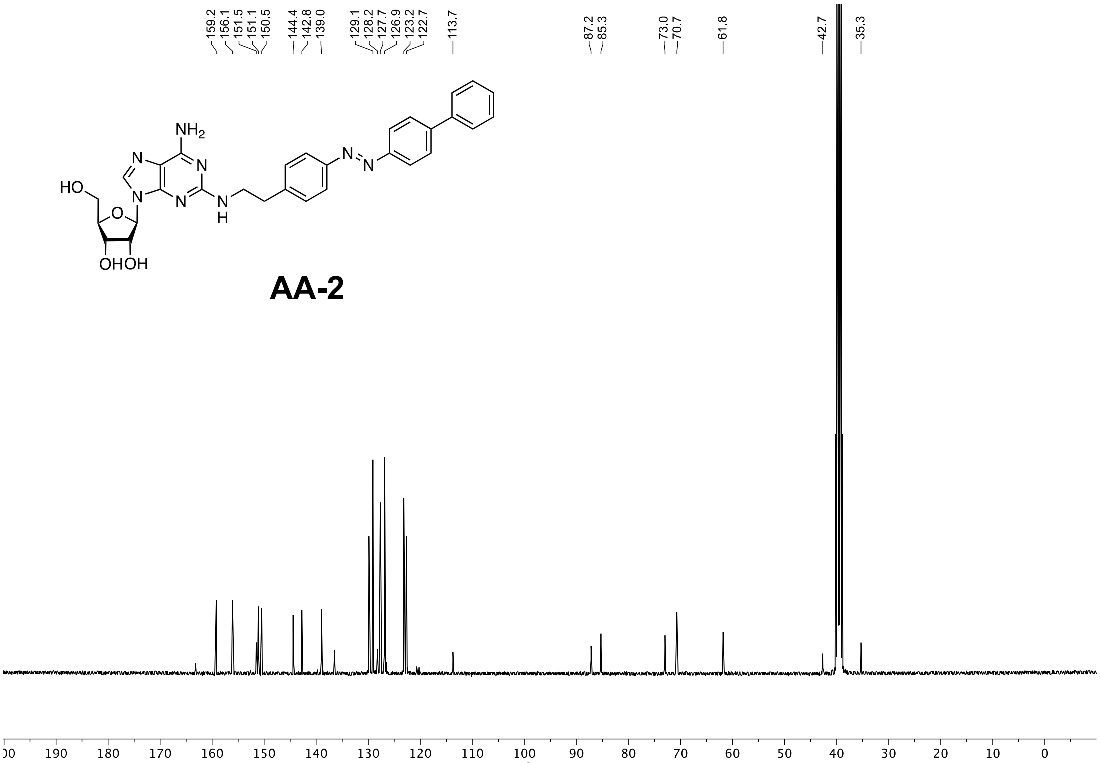
159.2 156.1 151.5 151.1 150.5 144.4 142.8 139.0 129.1 128.2 127.7 126.9 123.2 122.7 113.7 87.2 85.3 73.0 70.7 61.8 42.7 35.3
NH2
HO
OHOH
AA-2
f1 (ppm)

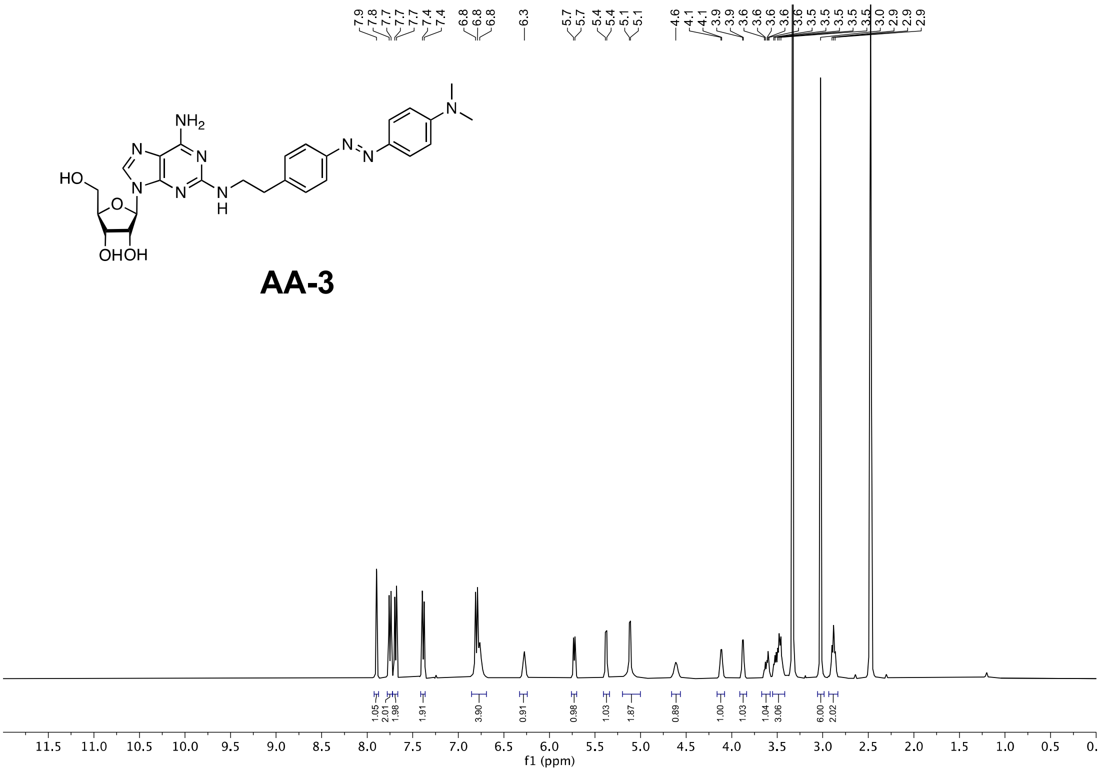
AA-3
f1 (ppm)

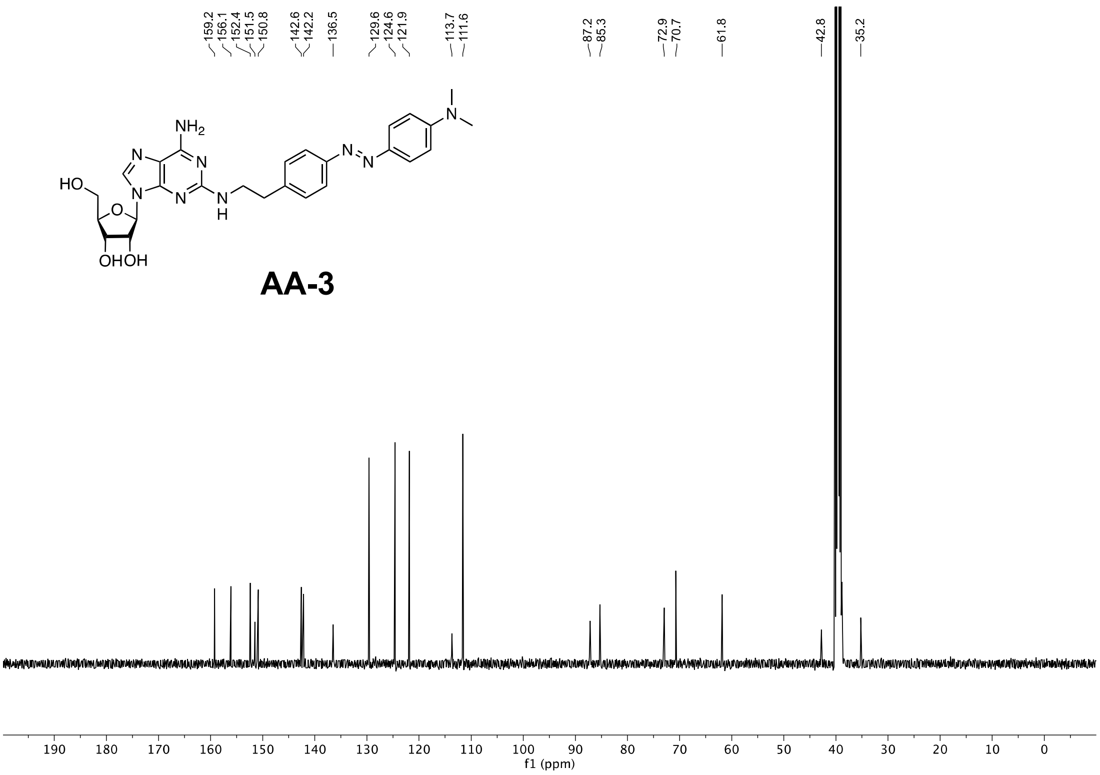
AA-3
f1 (ppm)

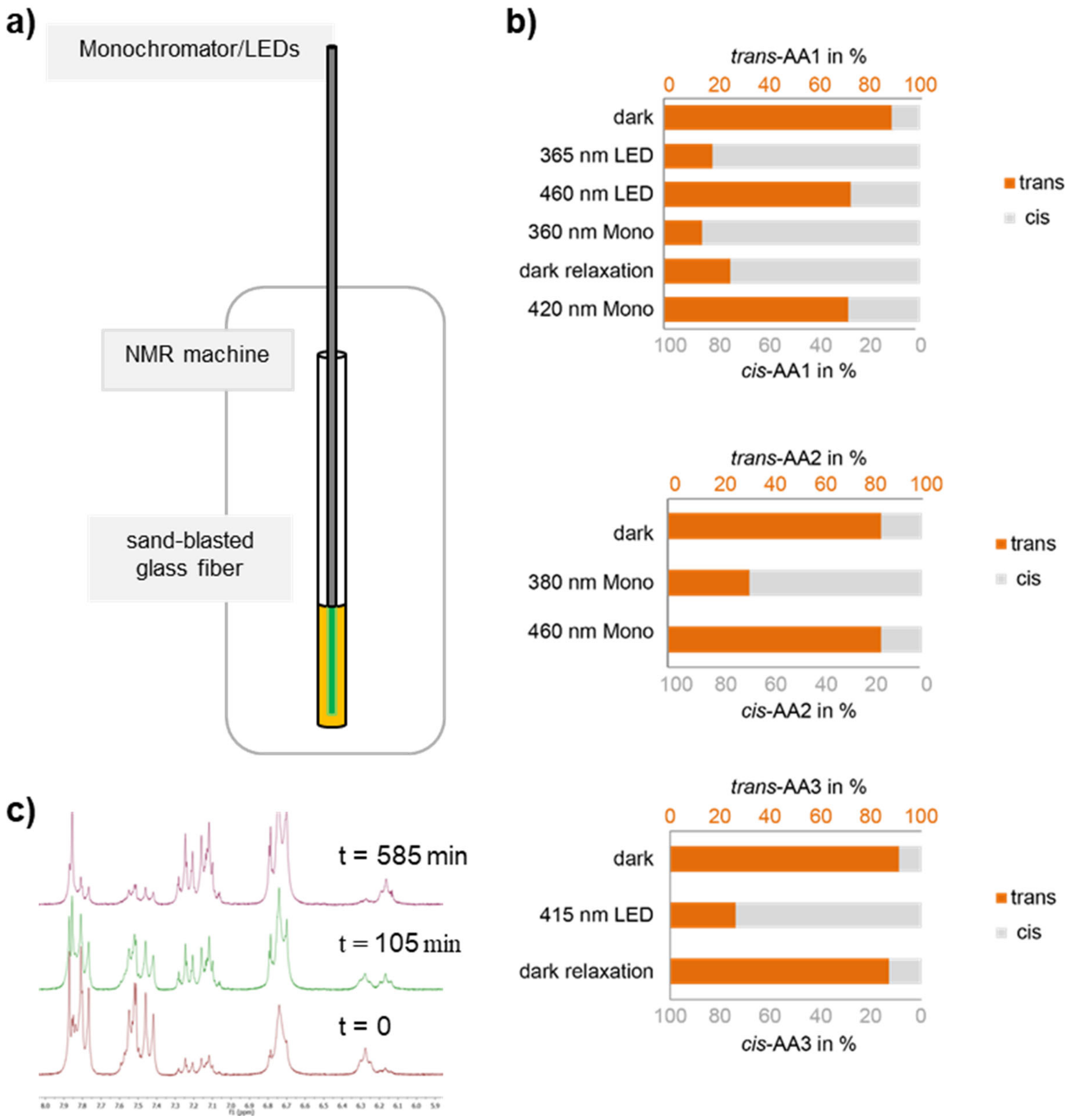

Figure S1. Determination of photostationary states (PSSs). a) Schematic depiction of the NMR with glass-fiber and light source to determine photostationary states by NMR. b) PSS-determination for all three AzoAdenosines by NMR. Ambient light: samples were wrapped in tin foil prior to measurement after preparation of NMR sample under ambient light conditions. Illumination: 12-14 h. Illumination: 12-14 h. Dark relaxation: no illumination. [a]: 12 h, [b]: 14h, [c]: for the red-shifted derivative, an illumination inside the NMR was not practical. The sample was irradiated outside the NMR and the maximum *cis*-content of the PSS was extrapolated. [d]: 30 min c) 1H-NMR (200 MHz) cutout, showing *trans* to *cis* isomerization over time.

Biological Data

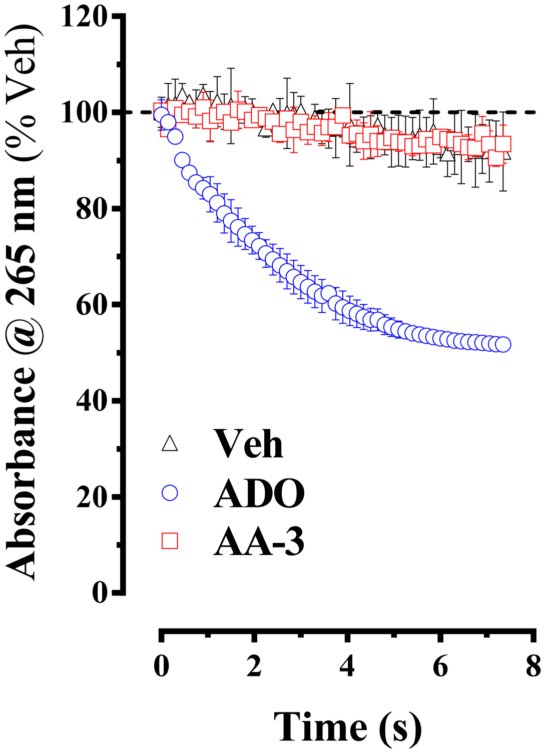

Figure S2. Time course of adenosine deaminase activity. Adenosine deaminase (ADA, 0.02 U/mL) activity was determined by measuring 265 nm absorbance reduction resulting from deamination of vehicle (Veh), adenosine (ADO, 100 mM) or **AA-3** (100 mM) over the time.

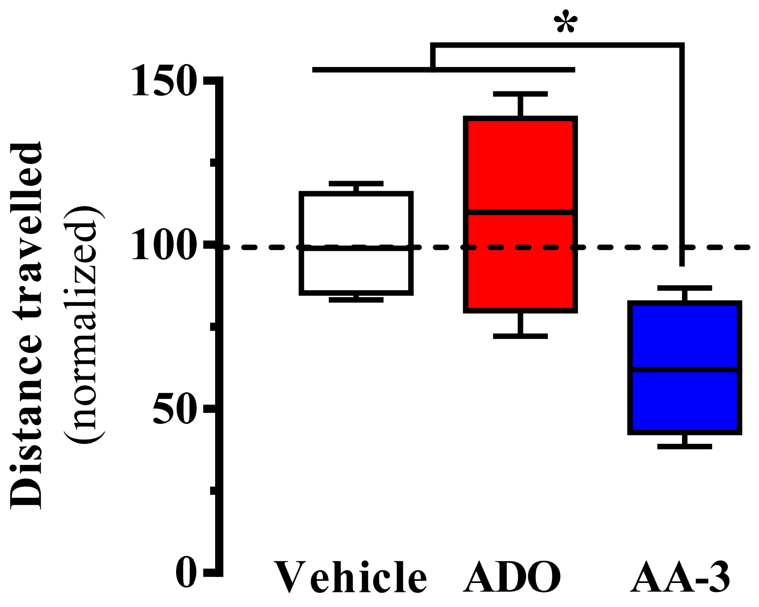

Figure S3. AA-3 induced locomotor activity. Locomotor activity was assessed by quantifying the total distance travelled of mice (n=5) systemically (i.p.) administered with vehicle (saline), adenosine (ADO, 10 mg/kg) or AzoAdenosine-3 (AA-3, 10 mg/kg) during 10 min. *P<0.05 and ***P < 0.001, one-way ANOVA with Dunnett's multiple comparison test using Veh as a control.

Table S1: Inhibition of binding of hot ligand to ARs by AA-1, AA-2 and AA-3 for A_1, A_{2A} and A_{2B} receptors.

Compound	Receptor	Hot ligand	Mean±SD% inhibition
AA-1	A_1	DPCPX	25.81±12.05
	A_{2A}	ZM241385	69.88±28.44
	A_{2B}	ZM241385	54.49±7.15
AA-2	A_1	DPCPX	2.92±6.19
	A_{2A}	ZM241385	62.30±23.77
	A_{2B}	ZM241385	25.19±12.53
AA-3	A_1	DPCPX	25.45±7.53
	A_{2A}	ZM241385	92.46±10.83
	A_{2B}	ZM241385	63.62±6.45

Table S2: Binding constants of AA-1, AA-2 and AA-3 for A_1 and A_{2A} receptors. mean±SD out of two experiments for A_{2A}. (A_1: $K_{i\,(CCPA)} = 0.50 \pm 0.06$, A_{2A}: $K_{i\,(ZM241385)} = 2.49 \pm 0.17$)

Compound	Receptor	Hot ligand	Ki (nM)
AA-1	A_1	DPCPX	> 10000
	A_{2A}	ZM241385	429±75
AA-2	A_1	DPCPX	> 10000
	A_{2A}	ZM241385	1418±480
AA-3	A_1	DPCPX	3023
	A_{2A}	ZM241385	509±120

References

(1) Schönberger, M. (2014) Developing Photoswitchable Ligands for Transmembrane Receptors. PhD Thesis, Ludwig-Maximilian-University, Munich.

(2) Hüll, K. (2019) Photopharmacology of ion channels, adenosine receptors and myosin-V. PhD Thesis, Ludwig-Maximilian-University, Munich.

(3) Besnard, J., Ruda, G. F., Setola, V., Abecassis, K., Rodriguiz, R. M., Huang, X. P., Norval, S., Sassano, M. F., Shin, A. I., Webster, L. A., Simeons, F. R. C., Stojanovski, L., Prat, A., Seidah, N. G., Constam, D. B., Bickerton, G. R., Read, K. D., Wetsel, W. C., Gilbert, I. H., Roth, B. L., and Hopkins, A. L. (2012) Automated design of ligands to polypharmacological profiles. *Nature 492*, 215–220.

(4) Gracia, E., Farré, D., Cortés, A., Ferrer-Costa, C., Orozco, M., Mallol, J., Lluís, C., Canela, E. I., McCormick, P. J., Franco, R., Fanelli, F., and Casadó, V. (2013) The catalytic site structural gate of adenosine deaminase allosterically modulates ligand binding to adenosine receptors. *FASEB J. 27*, 1048–1061.

(5) Clark, J. D., Gebhart, G. F., Gonder, J. C., Keeling, M. E., and Kohn, D. F. (1997) Special report: the 1996 guide for the care and use of laboratory animals. *ILAR J. 38*, 41–48.

(6) Rial, D., Castro, A. A., Machado, N., Garção, P., Gonçalves, F. Q., Silva, H. B., Tomé, Â. R., Köfalvi, A., Corti, O., Raisman-Vozari, R., Cunha, R. A., and Prediger, R. D. (2014) Behavioral phenotyping of parkin-deficient mice: looking for early preclinical features of Parkinson's disease. *PLoS One* (Tort, A. B. L., Ed.) *9*, e114216.

(7) López-Cano, M., Font, J., Llebaria, A., Fernández-Dueñas, V., and Ciruela, F. (2019) Optical Modulation of Metabotropic Glutamate Receptor Type 5 In Vivo Using a Photoactive Drug. *Methods Mol. Biol. 1947*, 351–359.

(8) Gómez-Santacana, X., Pittolo, S., Rovira, X., Lopez, M., Zussy, C., Dalton, J., Faucherre, A., Jopling, C., Pin, J., Ciruela, F., Goudet, C., Giraldo, J., Gorostiza, P., and Llebaria, A. (2017) Illuminating Phenylazopyridines To Photoswitch Metabotropic Glutamate Receptors: From the Flask to the Animals. *ACS Cent. Sci. 3*, 81–91.
